# Prognostic and Predictive Biomarkers in Non-Small Cell Lung Cancer Patients on Immunotherapy—The Role of Liquid Biopsy in Unraveling the Puzzle

**DOI:** 10.3390/cancers13071675

**Published:** 2021-04-02

**Authors:** Elien Augustus, Karen Zwaenepoel, Vasiliki Siozopoulou, Jo Raskin, Stephanie Jordaens, Geert Baggerman, Laure Sorber, Geert Roeyen, Marc Peeters, Patrick Pauwels

**Affiliations:** 1 Center for Oncological Research Antwerp (CORE), Integrated Personalized & Precision Oncology Network (IPPON), University of Antwerp (UAntwerp), 2610 Wilrijk, Belgium; Karen.Zwaenepoel@uza.be (K.Z.); Vasiliki.Siozopoulou@uza.be (V.S.); Stephanie.Jordaens@uantwerpen.be (S.J.); Laure.Sorber@uantwerpen.be (L.S.); Marc.Peeters@uza.be (M.P.); Patrick.Pauwels@uza.be (P.P.); 2 Laboratory of Pathological Anatomy, Antwerp University Hospital (UZA), 2650 Edegem, Belgium; 3 Department of Pulmonology and Thoracic Oncology, Antwerp University Hospital (UZA), 2650 Edegem, Belgium; Jo.Raskin@uza.be; 4 Centre for Proteomics, University of Antwerp (UAntwerp), 2020 Antwerpen, Belgium; Geert.Baggerman@uantwerpen.be; 5 Health Unit, Vlaamse Instelling voor Technologisch Onderzoek (VITO), 2400 Mol, Belgium; 6 Department of Hepato-Pancreato-Biliary, Endocrine and Transplantation Surgery, Antwerp University Hospital (UZA), 2650 Edegem, Belgium; Geert.Roeyen@uza.be; 7 Department of Oncology, Multidisciplinary Oncological Center Antwerp, Antwerp University Hospital (UZA), 2650 Edegem, Belgium

**Keywords:** liquid biopsy, non-small cell lung cancer, immunotherapy, molecular pathology

## Abstract

**Simple Summary:**

The introduction of immunotherapy modified the cancer treatment landscape, especially for non-small cell lung cancer (NSCLC). Unfortunately, only a subgroup of patients benefits from this therapy. Currently, the only validated companion diagnostic test for first-line immunotherapy in metastatic NSCLC patients is testing for programmed death ligand 1 (PD-L1) expression in tumor tissues. However, obtaining tumor tissue can be challenging and it puts the patient at risk. Liquid biopsy offers an alternative, less invasive approach to select NSCLC patients who would benefit from immunotherapy and to monitor patients during their disease course. Liquid biopsy allows repetitive sampling, which makes it a useful tool in clinical practice. In this review, we discuss the challenges and opportunities of several liquid biopsy-based prognostic and predictive biomarkers in NSCLC patients receiving immunotherapy.

**Abstract:**

In the last decade, immunotherapy has been one of the most important advances in the non-small cell lung cancer (NSCLC) treatment landscape. Nevertheless, only a subset of NSCLC patients benefits from it. Currently, the only Food and Drug Administration (FDA) approved diagnostic test for first-line immunotherapy in metastatic NSCLC patients uses tissue biopsies to determine the programmed death ligand 1 (PD-L1) status. However, obtaining tumor tissue is not always feasible and puts the patient at risk. Liquid biopsy, which refers to the tumor-derived material present in body fluids, offers an alternative approach. This less invasive technique gives real-time information on the tumor characteristics. This review addresses different promising liquid biopsy based biomarkers in NSCLC patients that enable the selection of patients who benefit from immunotherapy and the monitoring of patients during this therapy. The challenges and the opportunities of blood-based biomarkers such as cell-free DNA (cfDNA), circulating tumor cells (CTCs), exosomes, epigenetic signatures, microRNAs (miRNAs) and the T cell repertoire will be addressed. This review also focuses on the less-studied feces-based and breath-based biomarkers.

## 1. Introduction

With an estimated incidence of more than 2 million cases and approximately 1.8 million deaths annually, lung cancer is the leading cause of cancer-related death, worldwide [1]. Non-small cell lung cancer (NSCLC) accounts for approximately 85% of lung cancers [2]. At the time of diagnosis, locoregionally advanced or metastatic disease is present in more than 60% of the patients.

In the last decade, the treatment landscape and the prognosis of NSCLC patients have changed, resulting in a clinically meaningful improvement in survival and quality of life [3,4]. More specifically, targeted therapies gained a profound role in the management of NSCLC. Targeted therapy is a type of precision medicine, since cancer cells are targeted preferentially, resulting in less side effects than with traditional chemotherapy. However, only a subgroup of the NSCLC patients—harboring specific genetic alterations—benefits from this type of therapy [5]. More recently, immune checkpoint inhibitor agents targeting programmed cell death protein 1 (PD-1) or programmed cell death ligand 1 (PD-L1) have been established as a new treatment for advanced NSCLC patients. Of note is that a minority of NSCLC patients (< 20%) respond to this expensive therapy when administrated in monotherapy [6,7,8,9]. Immunotherapy is often given in combination with chemotherapy since several chemotherapeutical compounds appear to have the capacity to upregulate PD-L1 expression on cancer cells and to promote antitumor immunogenicity [10]. In 2018, the Food and Drug Administration (FDA) approved the combination of anti-PD-1 (pembrolizumab) with chemotherapy drugs (pemetrexed and carboplatin) to treat metastatic NSCLC [6]. 

Hence, it is crucial to identify predictive biomarkers for NSCLC patients on immunotherapy to enable the selection of patients that will benefit from this therapy. For the early detection of resistance to anti-PD-1/PD-L1 therapy, the identification of biomarkers which allow monitoring of the NSCLC patients during therapy is important [11]. 

Within this context, the interest in the liquid biopsy field started to grow. Liquid biopsy refers to tumor-derived material that is present in body fluids. Recently, liquid biopsy has become an attractive approach since it is a less invasive, cost-effective technique which gives real-time information on the tumor characteristics [12]. In this light, in 2016 the Cobas EGFR Mutation Test v2 (Roche) became the first liquid biopsy assay that obtained the approval of the FDA for the identification of NSCLC patients eligible for epidermal growth factor receptor (EGFR)-based targeted therapy [13]. 

In this review we will: (i) discuss which components of a liquid biopsy sample can be used as a predictive or prognostic biomarker in NSCLC patients on immunotherapy, and (ii) highlight the challenges and clinical applicability of each biomarker.

## 2. Liquid Biopsy

The treatment strategy for metastatic NSCLC patients is largely based on two parameters: the presence of specific molecular or genomic aberrations within the tumor tissue, and imaging [14]. Tissue analysis, however, faces some limitations. The procedure to obtain a tissue biopsy can put the patient at risk, sometimes it is not possible to obtain adequate tumor tissue due to the location of the tumor and studying tumor heterogeneity requires multiple biopsies which is difficult due to ethical and practical considerations [15,16,17]. 

Evaluating treatment response by imaging can be difficult, particularly when dealing with immunotherapy. Pseudoprogression is characterized by radiologic enlargement of the tumor mass. It is caused by the infiltration of leukocytes and associated with favorable a long-term survival. Pseudoprogression is reported in up to 6% of metastatic NSCLC treated with anti-PD-1/PD-L1 therapy. In 2009, the Response Evaluation Criteria in Solid Tumors (RECIST) scoring was adapted for the radiographic monitoring of immunotherapy receiving patients (iRECIST) in an attempt to overcome this issue [18,19].

**Figure 1:**
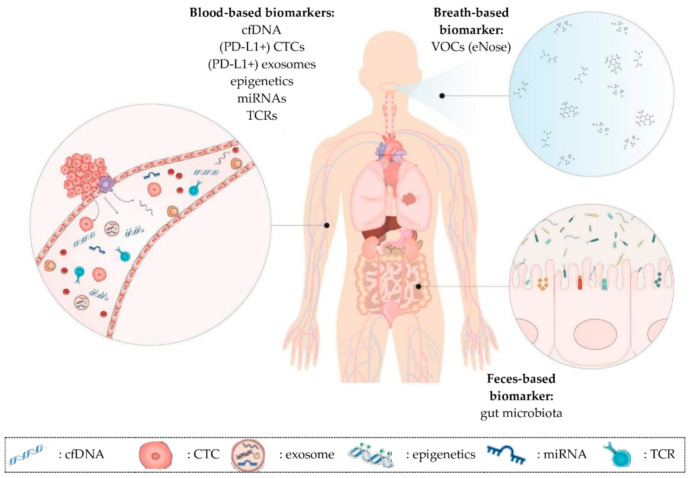
Overview of promising prognostic and predictive minimally invasive biomarkers in NSCLC patients on immunotherapy discussed in this review. (1) Blood-based biomarkers such as cfDNA, CTCs, exosomes, epigenetic signatures, miRNAs and TCR repertoire. These biomarkers can also be present in other liquids than blood (e.g., urine). (2) Breath-based biomarkers such as VOCs. (3) Feces-based biomarkers such as the gut microbiota. cfDNA: cell-free DNA; CTCs: circulating tumor cells; miRNAs: microRNAs; TCR: T cell receptor; VOCs: volatile organic compounds.

Liquid biopsies might help to solve the issues regarding the selection and monitoring of NSCLC patients since it enables repetitive sampling. It allows the analysis of cancer-associated biomarkers in biological fluids such as blood, urine or saliva. Interestingly, even in feces and breath potential biomarkers can be detected. A liquid biopsy sample consists of different (circulating) components derived from cancer as well as from healthy tissue. In this review, the characteristics of the following components will be discussed: circulating cell-free DNA (cfDNA), circulating tumor cells (CTCs), extracellular vesicles (EVs), epigenetic signatures, microRNA (miRNA), volatile organic compounds (VOCs) and the gut microbiota (Table 1, Figure 1) [20,21].

Despite the potential benefits, major hurdles need to be overcome. Liquid biopsy-based biomarkers often lack sensitivity and specificity, especially in patients with localized tumors [22]. Furthermore, workflows need to be standardized and protocols need to be harmonized. Another hurdle is the lack of reimbursement for liquid biopsy-based tests in some countries [23,24]. Ongoing clinical trials in NSCLC patients on immunotherapy address these challenges and will give us more insight on the future of this technique (Table 2).

**Table 1:** Summary of characteristics of different blood-based biomarkers. The different advantages and disadvantages of the blood-based biomarkers, that favor or disfavor their use in clinical settings, are shown in the table. BEAMing: beads, emulsions, amplification and magnetics; cfDNA: circulating cell-free DNA; bTMB: blood tumor mutational burden; EpCAM: epithelial cell adhesion molecule; miRNA: microRNA; NGS: next-generation sequencing; NSCLC: non-small cell lung cancer; PD-L1: programmed death-ligand 1; TCR: T cell repertoire.

Type	Isolation Technique	Advantages	Disadvantages	Promising Biomarker	References
Cell-free DNA	Magnetic beads or spin column based	- Easy and well-established isolation procedures - Allows real-time monitoring	- Short half-life: 16 min–2.5 h - Not stable in circulation - Long turn-around time NGS based tests - Sensitive detection methods required	bTMB and levels of cfDNA for cancer diagnosis and treatment evaluation	[17,25,26,27,28,29,30,31,32,33,34,35,36,37,38,39,40,41,42,43,44,45,46,47,48,49,50,51,52,53]
Circulating tumor cells	Antigen-dependent (e.g., EpCAM) or size and deformability based method	- Provides transcriptomic, genomic and proteomic information	- Short half-life: 1–2.4 h - Not stable in circulation	PD-L1 analysis status to predict response on treatment	[15,25,54,55,56,57,58,59,60,61,62,63,64]
		- Intact viable cells	- Low abundance in NSCLC		
			- Isolation procedures requires high expertise and dedicated equipment		
Exosomes	Based on physical or biological properties of exosomes, immune-mediated isolation, sucrose gradient method, ultra-centrifugation	- Provides information about tumor’s biologic profile, growth rate, metastatic capacity and drug resistance - Abundant	- Isolation can be time-consuming and can alter exosome structure - Difficult to detect due to small size	Exosomal PD-L1 status to predict response on therapy	[25,65,66,67,68,69,70,71,72,73,74]
Epigenetics and miRNA	cfDNA isolation followed by methylation specific PCR or BEAMing	- Methylation makes cfDNA more stable - Allows real-time monitoring	- Sensitive detection methods required	Methylation status of genes (signature) for cancer diagnosis and treatment evaluation	[75,76,77,78,79,80,81,82,83,84,85,86,87]
T cell repertoire	Density centrifugation followed by flowcytometry	- Easy isolation - Intact viable cells	- Clustering of functionally different clones causing false positive results	The frequency, diversity and clonality of TCRs for cancer diagnosis and treatment evaluation	[41,57,88,89,90,91,92]

## 3. Cell-Free DNA

cfDNA is currently the material with the greatest potential in clinical practice [25]. The term cfDNA refers to both the encapsulated DNA (in circulating vesicles) as well as the non-encapsulated free DNA. cfDNA is mostly studied in blood samples, but is also present in other body fluids such as urine, saliva and cerebrospinal fluid. Its half-life ranges from 16 min to 2.5 h, due to rapid clearance by the circulation through the kidneys, liver and spleen.

cfDNA can be derived from healthy cells, as well as from tumor cells. The fraction originated from tumor cells is named circulating cell-free tumor DNA (cf tumor DNA). Cf tumor DNA is released into the peripheral blood by three main mechanisms: apoptosis, necrosis and active secretion from EVs and CTCs. The fraction of cfDNA contributed from the tumor varies greatly, between 0.01% and more than 90% [17]. The levels of cf tumor DNA are influenced by tumor burden and other factors such as tumor location, vascularity and cellular turnover [26]. Fortunately, sensitive detection platforms like beads, emulsions, amplification and magnetics (BEAMing), digital droplet PCR (ddPCR), MassARRAY and specialized next-generation sequencing (NGS) techniques make it possible to detect low concentrations of cf tumor DNA [27]. 

cfDNA fragments derived from healthy cells—often referred to as genomic DNA (gDNA) contamination—stem from apoptotic or necrotic cells, particularly white blood cells which have a limited survival time. The concentration of cfDNA can be influenced by certain conditions such as inflammation, infection and even exercise [25]. For qualitative analysis of cf tumor DNA, it is important to reduce the concentration of gDNA contamination to an absolute minimum. In this context, the use of an appropriate cfDNA extraction kit which favors the isolation of cf tumor DNA fragments is crucial [28]. 

### 3.1. Blood Tumor Mutational Burden

Tumor mutational burden (TMB) can be defined as the total number of somatic non-synonymous mutations per mega base (Mb) of the genome examined. The presence of these mutations can lead to the formation of neoantigens which are recognized by the immune system as non-self, resulting in the activation of the antitumor immune response. This hypothesis suggests that NSCLC patients with a high TMB could potentially benefit from immunotherapy [29]. Notably, a high mutational load does not mean that a high number of neoantigens will be expressed on the cancer cell surface [30]. There is also evidence that the quality of neoantigens (= an elevated immune-recognition potential) is more important than the quantity of neoantigens [31,32]. The number of somatic mutations varies between different cancer types. NSCLC has one of the highest mutation frequencies (0.1 to 100 mutations/Mb), particularly in smokers [33,34]. TMB in tumors is usually evaluated using NGS-based approaches (including whole exome sequencing (WES) and comprehensive genomic profiling (CGP)) [35,36] and seems to correlate with gender, since it is higher in men than in women [37]. 

Herbst R.S. et al. retrospectively investigated the association between tissue TMB (tTMB) and the clinical benefit with pembrolizumab monotherapy observed in previously treated (KEYNOTE-010, NCT01905657) or treatment naïve (KEYNOTE-042, NCT02220894) PD-L1+ NSCLC patients. In both trials, improvements in overall survival (OS) as well as in progression-free survival (PFS) were generally observed for patients treated with pembrolizumab with high tTMB (≥ 175) [38]. Several research groups studied the possibility to use blood (cf tumor DNA) instead of tumor tissue to determine TMB. The group of Wang et al.—who enrolled 48 patients with advanced NSCLC treated with anti PD-(L)1 therapy—observed that blood TMB (bTMB) correlated well with tTMB calculated by WES [39]. Similar results were obtained in larger study cohorts. The randomized and retrospective POPLAR (NCT01903993, *n* = 287) and OAK (NCT02008227, *n* = 1.225) clinical trials both compared atezolizumab with the standard of care, docetaxel, in NSCLC patients. In these clinical trials, tTMB and bTMB obtained from pre-treatment plasma from the same patients were compared. A positive correlation between tTMB and bTMB in NSCLC patients was observed (Spearman rank correlation: 0.64; 95% confidence interval (CI): 0.56–0.71) [40]. 

In this context, determining a cut-off point for bTMB was essential. The POPLAR trial demonstrated that bTMB  ≥  16 is a clinically meaningful and technically robust cut-off point in NSCLC patients. This observation was confirmed by the OAK trial [40,41]. Interestingly, NSCLC patients on immunotherapy with high bTMB were more likely to respond to therapy. Patients from the OAK trial with bTMB  ≥  16 obtained a significant PFS benefit (hazard ratio (HR): 0.65 (95% CI: 0.47–0.92); *p*  =  0.013) when treated with atezolizumab versus docetaxel [40]. Similar results were observed by Wang et al. [39]. Another clinical trial (B-F1RST, NCT02848651, *n* = 152) also showed a correlation between a high bTMB and atezolizumab response in NSCLC patients, using the same cut-off for bTMB as in the POPLAR and OAK studies [41]. 

These data suggest that bTMB might be a promising prognostic biomarker for NSCLC patients on immunotherapy. In this light, the FDA approved two plasma-based NGS assays for the measurement of bTMB, namely the Guardant Health (GH) Omni (500 genes, 2.1 Mb) and the Foundation Medicine (FMI) bTMB (394 genes, 1.14 Mb) panels in 2020 [42]. 

However, these promising results are in contrast with the preliminary data of the Checkmake 227 clinical trial (NCT02477826, *n* = 679). The results of this randomized trial showed a similar degree of OS benefit in patients who received nivolumab plus ipilimumab, regardless of whether they had a high or low bTMB (≥10 vs. <10 mutations per Mb, respectively) [43]. In addition, Paz-Ares et al. studied patients with non-squamous NSCLC (KEYNOTE-021 trial (NCT02039674) and KEYNOTE-189 trial (NCT02578680)) and patients with squamous NSCLC (KEYNOTE-407 trial (NCT02775435)). They found that tTMB was not significantly associated with the efficacy of first-line pembrolizumab plus platinum-based chemotherapy or with chemotherapy alone in NSCLC [44].

TMB studies the somatic non-synonymous mutations present in a Mb. Interestingly, some studies revealed that single point mutations can also be used to monitor NSCLC patients treated with immunotherapy. Guibert et al. showed that the presence of a *phosphatase and tensin homolog (PTEN)* or a *serine/threonine kinase 11 (STK11)* mutation was correlated with early progression in NSCLC patients receiving anti PD-1 immunotherapy. In contrast, transversion mutations (substitution of a purine by a pyrimidine or vice versa) in the *Ki-ras2 Kirsten rat sarcoma viral oncogene homolog (KRAS)* gene and *tumor suppressor gene TP53* (also known as p53) alone predicted better outcomes [45]. 

However, another study reported that NSCLC patients on anti PD-1 immunotherapy who harbored co-mutations with *STK11* and *KRAS* (*n* = 36) had longer OS in comparison to patients who harbored *STK11* mutations alone (13.6 ± 3.4 months, *p* = 0.049, *n* = 37). They further investigated the population-specific factors that influenced the survival of the cohort with *STK11/KRAS* mutations. The group revealed that NSCLC patients with both mutations often were: (i) older at diagnosis, (ii) more likely to have received nivolumab (as compared to pembrolizumab and atezolizumab), (iii) more likely to have longer smoking histories and (iv) harboring more targetable mutations [46]. Hence, caution is recommended, since controversial results are published regarding the presence of mutations in these genes vs. the response to immunotherapy (mutations in *KRAS* or *STK11* gene alone vs. mutations in both genes).

Interestingly, Sun et al.—who enrolled 240 patients—observed that NSCLC patients who harbored *AT-rich interacting domain-containing protein 1A gene (ARID1A)* mutations or *AT-rich interacting domain-containing protein 1B gene (ARID1B)* mutations had a beneficial response to anti PD-(L)1 immunotherapy and a prolonged PFS [47]. Other mutations also showed promising results. The OAK and POPLAR clinical trials showed that mutations in the *kelch-like ECH-associated protein 1 (KEAP1)* and *nuclear factor erythroid-2-related factor-2 (NFE2L2)* genes were associated with poorer OS and PFS (OS: HR = 1.7, *p* < 0.001; PFS: HR = 1.4, *p* < 0.001) in NSCLC patients receiving immunotherapy [48].

We found conflicting results regarding TMB, which is not a perfect biomarker to monitor NSCLC patients on immunotherapy. Furthermore, NGS is expensive and sequencing results can vary between laboratories as a function of the use of the cut-off for TMB-high, panel size, composition and bioinformatics pipelines. NGS also has a long turn-around time. Based on the challenges and the contradictory results concerning bTMB as a biomarker, further understanding is warranted before the integration of this factor into clinical practice [43,49,50]. 

### 3.2. Levels of cfDNA

Interestingly, the levels of cfDNA in the blood circulation can also be used to monitor NSCLC patients on immunotherapy. Cabel et al. investigated the role of the concentration of cfDNA in plasma samples of patients with NSCLC, melanoma or colorectal cancer, (CRC) treated with nivolumab or pembrolizumab monotherapy. They observed a significant correlation between synchronous changes in cf tumor DNA levels and tumor size, eight weeks after the first administration of immunotherapy. They reported that patients with undetectable cf tumor DNA levels at week 8 had significantly better PFS and OS than patients with persistently detectable cf tumor DNA [51]. Other research groups confirmed these findings. Giroux Leprieur et al. studied advanced NSCLC patients during nivolumab treatment. A high cf tumor DNA concentration at two months (first tumor evaluation) and an increase in concentration compared to the baseline were associated with a poor response and no long-term clinical benefit. Low cf tumor DNA concentrations at two months were associated with a long-term benefit of nivolumab [52]. Goldberg et al. reported that a drop in cf tumor DNA level was an early marker of therapeutic efficacy and a predictor for a prolonged survival in NSCLC patients treated with immune checkpoint inhibitors [53]. It is important to note that only a small number of participants (≤28 participants) were included in all three studies. Despite the fact that these data need to be confirmed in larger study cohorts, measuring the levels of cfDNA is a potential prognostic biomarker to monitor NSCLC patients on immunotherapy.

## 4. CTCs

CTCs are cancer cells that are detached from the primary tumor or metastatic tumors. They are part of the metastasis process [54]. By passive shedding and/or intravasation (with epithelial–mesenchymal transition), CTCs get into the bloodstream [15]. CTCs can provide transcriptomic, genomic and proteomic information. They can be observed non-clustered as a single cell, or as a cluster of multiple CTCs. These aggregates which are comprised of a minimum of two CTCs are characterized by a significantly enhanced metastatic potential in comparison to single CTCs. The presence of CTC clusters in the blood circulation is associated with a poor prognosis in NSCLC patients [55]. CTCs are less stable than cfDNA and they have short half-life (1–2.4 h). Since the proportion of CTCs in the bloodstream is very low in NSCLC patients, dedicated equipment and high expertise is needed [56]. Currently, there are several methods to detect and isolate CTCs that take different aspects into account. The CellSearch^®^ system (Menarini), the Epic platform (Epic sciences) and the GILUPI CellCollector (GILUPI) are based on the presence of specific antigens (e.g., epithelial cell adhesion molecule (EpCAM)) on the surface of the CTCs. In addition, antigen-independent methods have also been developed to isolate CTCs based on their size and deformability, such as Parsortix (Angle), Isolation by SizE of Tumor cells or in short ISET (Rarecells Diagnostics), Vortex VTX-1 (Vortex Bioscience) and the ClearCell FX device (Biolidics) [25,57]. Interestingly, CTCs can be frequently detected in metastatic breast cancer (BC) patients. Approximately 70% of metastatic BC patients exhibit no less than one CTC per 7.5 mL of blood and 50% exhibit no less than five CTCs per 7.5 mL [58]. The FDA even approved the CellSearch^®^ for CTC enumeration in BC patients [59]. CTCs are considered to have been derived from more than one tumor site, resulting in a better global representation of PD-L1 expression than tissue samples [57].

The studies evaluating the concordance between PD-L1 expression by CTCs and PD-L1 expression in tissue showed controversial results. Ilie et al. compared the PD-L1 status of CTCs, using the ISET method, with the PD-L1 status in tissue in NSCLC patients. In this study, PD-L1 expression on CTCs and matched tissue biopsies were well correlated (93%) [60]. Guibert et al. studied 96 NSCLC patients treated with chemotherapy followed by immunotherapy. In contrast, they found that CTCs were more frequently PD-L1-positive than tissue (83% vs. 41%). Consequently, no correlation between tissue and CTC PD-L1 expression was observed. The ISET platform was used for the isolation of CTCs. Other research groups reported similar results as Guibert and colleagues. However, different PD-L1 analyzing methods and antibodies were used [61,62,63]. 

Interestingly, PD-L1-positive CTCs are shown to be a promising predictive biomarker in NSCLC patients receiving immunotherapy. Guibert et al. also presented that a higher baseline PD-L1+ CTC number (≥1%) was observed in NSCLC patients who did not respond to therapy (PFS < 6 months). Importantly, the presence of pre-treatment PD-L1+ CTCs was not significantly correlated with the clinical outcomes [61]. Another study of 24 metastatic NSCLC patients on nivolumab displayed that the presence of CTCs and the expression of PD-L1 on their surface at baseline and at 3 months of treatment were associated with poor patient outcome [64]. 

Due to the lack of standardized methods to detect and analyze CTCs, the results of these studies must be interpreted with caution. Furthermore, issues regarding the contradictory results about the use of CTCs as a biomarker need to be addressed. Fortunately, different clinical trials are ongoing at the moment which address these challenges. The use of CTC to determine the PD-L1 status is a potential biomarker in NSCLC patients to predict their response to immunotherapy.

## 5. Tumor-Derived Exosomes

EVs are lipid-bound vesicles secreted by cells into the extracellular space. EVs have a half-life from approximately a number of minutes to 6 h [65]. They have a small size (30–1.000 nm), therefore isolation of the vesicles is difficult. However, several techniques allow the extraction of EVs such as: (sucrose density gradient) ultracentrifugation, microfiltration, gel filtration, density gradient centrifugation (OptiPrep) and size-exclusive chromatography. Importantly, the isolation of EVs can be time-consuming and can alter the structure of the vesicles [66]. The three main subtypes of EVs, which differ in their biogenesis, release pathways, size, content and function, are: micro vesicles, exosomes and apoptotic bodies [67]. The most studied subtype is exosomes. 

Exosomes are abundant nanosized particles with lipid bilayer membranes which have a size between 50 and 150 nm. They are secreted by several cell types, including tumor, immune and lymphoid cells, or they can be derived from the stroma [68]. Exosomes released from stromal cells are able to stimulate nearby tumor cells to metastasize. They also promote tumor cell proliferation and inhibit their apoptosis. Tumor cells, as well as immune cells, secrete immunologically active exosomes that affect the antitumor activities of other immune cells, causing a favorable environment for the tumor [67]. 

In this review, we will focus on tumor-derived exosomes (TEX). TEX—which only account for a small number of the total amount of exosomes—are involved in the development of cancer, the formation of metastasis and disease progression. They play an important role in cell–cell communication, and it is known that TEX are associated with the development of resistance to chemotherapy. TEX also play a role in the emergence of the radiation-induced bystander effect, which is a phenomenon wherein non-targeted cells exhibit the effects of radiation [69,70]. Furthermore, TEX have the ability to inhibit immune cell proliferation. They can also induce apoptosis (or suppression) of immune cells such as CD8+ T cells. Hence, TEX influence the sensitivity of tumor cells to immunotherapy [25,67,71].

In this context, a few research groups investigated the role that TEX might play in the selection of NSCLC patients who might benefit from immunotherapy. A study of 24 patients with lung cancer before surgery presented a correlation between the number of plasma-derived PD-L1+ exosomes and the PD-L1 expression level in the tumor tissue [72]. In contrast, Li et al., who enrolled 85 NSCLC patients, did not find a correlation between the PD-L1 status in exosomes versus in tumor tissue [73]. Gunasekaran et al. presented that PD-L1-positive TEX can be used as a predictive biomarker for NSCLC patients who are treated with immunotherapy. They observed a trend towards PD-L1 reduction in patients who responded to immunotherapy. However, this difference was not statistically significant. Interestingly, they suggested that the dynamic changes of exosomal PD-L1 (pre-treatment vs. 8 weeks of treatment) predicted the clinical outcome in terms of both PFS and OS in patients treated with immunotherapy. Again, here, a limitation of the study was that only 25 NSCLC patients were included [74]. 

Several challenges should be overcome to make the implementation of EVs in the clinical practice feasible. The first and foremost challenge consists of the standardization of the methodologies for EV isolation and purification [71]. Nevertheless, evaluating the PD-L1 status based on exosomes is a promising biomarker for the selection of NSCLC patients that may benefit from immunotherapy.

## 6. Epigenetics and microRNAs

The interest in using epigenetic-based markers and miRNAs to monitor NSCLC patients on immunotherapy has recently started to grow. Epigenetic components such as DNA methylation, histone modifications, nucleosome positioning and non-coding RNAs, specifically miRNAs, largely influence tumor cells’ functioning [75]. 

Hypermethylation (of tumor suppressor genes) was shown to contribute to carcinogenesis. One of the main advantages of DNA methylation alterations, compared to other potential diagnostic biomarkers, is that they are remarkably stable, and generally occur early during carcinogenesis. To obtain methylated DNA, first, cfDNA isolation needs to be performed, followed by methylation-specific PCR or BEAMing [76].

Different research groups analyzed the possibility of using methylation signatures or patterns as biomarkers to monitor NSCLC patients treated with immunotherapy. Druisseaux et al. established an epigenomic profile based on a microarray DNA methylation signature (EPIMMUNE) in a set of tissue biopsies from IV stage NSCLC patients treated with nivolumab or pembrolizumab. They showed that the EPIMMUNE signature was associated with improved PFS and OS. However, no association was found between the EPIMMUNE signature and PD-L1 expression [77]. Cho et al. confirmed that methylation patterns could be used for the monitoring of NSCLC patients during therapy. They studied differentially methylated regions overlapping promoters (pDMRs) or enhancers (eDMRs) in tissue biopsies between responders and non-responders to immunotherapy. Interestingly, they identified 1.007 pDMRs and 607 eDMRs which were associated with the anti-PD-1 response [78,79]. 

In this context, we will highlight specific genes that might be a promising biomarker to monitor NSCLC patients treated with immunotherapy. First, the methylation status of the *transcription factor T cell-related forkhead box P1 (FOXP1)* was significantly associated with improved PFS and OS in NSCLC patients following immunotherapy [77]. Furthermore, hypomethylated pDMRs of the *Cytohesin 1 Interacting Protein (CYTIP)* and the *TNF superfamily member 8 (TNFSF8)* could predict response in NSCLC patients on anti-PD-1 therapy. In fact, the authors even suggested that their ability to predict the clinical outcome was superior to that of the commonly used biomarker PD-L1 [78,79]. 

miRNAs are small, single-stranded non-coding RNA sequences of approximately 18–22 nucleotides that are regulators of gene expression, that can be derived from either cancer cells or immune cells [80,81]. They are known to have important roles at post-transcriptional and translational levels [82]. Analysis of circulating miRNAs can be accomplished with quantitative real time-PCR (RT-PRCR), microarray or deep sequencing after initial ultracentrifugation [83]. 

Studies revealed that miRNAs play an important role in the regulation of PD-L1. Cortez et al. found that PD-L1 was regulated by p53 via miR-34 which regulated (in)directly the expression of several immune checkpoints [84]. Furthermore, miRNAs were associated with the clinical outcome of NSCLC patients who were treated with immunotherapy. Fan et al. presented that NSCLC patients who responded to nivolumab (*n* = 17) had long-term increased expression levels of miR-93, −138-5p, −200, −27a, −424, −34a, −28, −106b, −193a-3p and −181a from pre-treatment to post-treatment in their serum versus non-responders (*n* = 17). They reported that the high expression of these ten miRNA patterns was significantly correlated with an improvement in OS and PFS [82]. Another study of Boeri et al. displayed that a miRNA signature classifier (MSC) composed of 24 miRNAs could distinguish NSCLC patients (*n* = 140) that would benefit from anti PD-L1 immunotherapy from patients who would not. The MSC risk level was associated with the overall response rate (ORR (*p* = 0.0009), PFS (multivariate HR = 0.31; 95% CI: 0.17–0.56; *p* = 0.0001) and OS (multivariate HR = 0.33; 95% CI: 0.18–0.59; *p* = 0.0002)) [85]. In this light, Epigenomics AG developed two liquid biopsy-based tests to evaluate the methylation status of certain genes for early detection of CRC or lung cancer. The Epi proLung assay can be used to detect lung cancer by the determination of the methylation status of the *short stature homeobox 2 (SHOX2)* and the *prostaglandin E receptor 4 (PTGER4)* gene [86]. The Epi proColon assay, which detects the methylation patterns of the gene *septin 9 (SEPT9)* was approved by the FDA for CRC screening [87]. 

Despite the fact that a few technical issues need to be resolved to increase the sensitivity of this potential biomarker, the use of epigenetics and miRNAs is a promising tool to monitor NSCLC patients following immunotherapy treatment. Fortunately, several sensitive detection platforms are already commercially available.

## 7. T Cell Receptor Repertoire

T cell receptors (TCRs) are antigen-specific receptors that are present at the cell surface of T lymphocytes and play an important role in the immune response [41]. Isolation of T cells from patients’ blood can easily be done by density centrifugation. The biggest advantage of this technique is that T cells are still in a fully functional state after isolation. Afterwards, flow cytometry is often performed to select CD4+ and CD8+ cells. In the majority of T cells, the TCR consists of one alpha and one beta chain. Each chain contains three complementarity-determining regions (CDRs) which are hypervariable regions. The CDR3 region variability is generated by genetic recombination and is consequently unique to each TCR. Sequencing this specific region allows the determination of the TCR repertoire which contains a diversity of certain T cell clones that are responsible for anti-tumor immunity [57,88,89]. Characterization of these specific T cell clones may lead to future studies which may expand these clones in a personalized approach to potentially lengthen the duration of the sustained response to immunotherapy in the patient [90].

The possibility to use the clonality and the diversity of the TCR repertoire as a prognostic biomarker for NSCLC patients on immunotherapy was studied by several research groups. Han et al. collected blood samples from 40 NSCLC patients receiving anti PD-(L)1 immunotherapy. They observed that NSCLC patients with a high PD-1-positive CD8+ TCR diversity before treatment had a better clinical outcome in comparison to patients with a low diversity (6.4 versus 2.5 months). In addition, patients with an increased PD-1-positive CD8+ TCR clonality after immunotherapy showed longer PFS than patients with decreased clonality (7.3 versus 2.6 months [91]. In 2020, Yamauchi et al. focused on the role of the CX3C chemokine receptor 1 (CX3CR1)—which is a marker of T cell differentiation—in predicting the response to immunotherapy. They presented that the TCR frequency and clonality of the peripheral CX3CR1-positive CD8+ T cell subset (which included an enriched repertoire of tumor-specific and tumor-infiltrating CD8+ T cells) was increased in tumor-bearing mice who responded to immunotherapy. Furthermore, they observed a correlation between a positive clinical outcome and an increase in the frequency of the CX3CR1-positive subset in circulating CD8+ T cells based on the analysis of peripheral blood mononuclear cell (PBMC) samples from 36 NSCLC patients treated with nivolumab or pembrolizumab [92].

However, a few hurdles need to be resolved before the implementation in clinic is feasible. Challenges regarding the high rate of false positives, caused by the clustering of functionally different clones or the presence of artificial clones, need to be addressed. Nevertheless, TCR diversity and clonality might serve as a biomarker to monitor the response of NSCLC patients on immunotherapy [91].

## 8. Gut Metabolism

Feces are gaining a more profound role in the liquid biopsy field. Specifically, the interest in using the gut microbiota as a biomarker to monitor NSCLC patients treated with immunotherapy has started to grow. Even though it can be questioned whether feces are a body “liquid”, we will consider it as a liquid biopsy in concurrence with other publications [93,94]. Feces can be used to study the gut microbiota, which is composed of bacteria, protists, fungi and viruses. Interestingly, it appears that metabolic changes occurring in the gut microbiota metabolome are associated with the response to immunotherapy in NSCLC [95].

Botticelli et al. characterized the metabolomic profiling of the gut microbiota of eleven NSCLC patients receiving nivolumab. They reported that some gut microbiota, such as 2-pentanone (ketone) and tridecane (alkane), were significantly associated with early progression. In contrast, short-chain fatty acids (i.e., propionate, butyrate), lysine and nicotinic acid were significantly associated with a long-term beneficial outcome [96]. Jin et al. revealed that the diversity of the microbiome also affected the clinical outcome on immunotherapy. The research group enrolled 37 Chinese patients with advanced NSCLC treated with nivolumab. They showed that patients with a high microbiome diversity had a significantly prolonged PFS in comparison to patients with a low diversity. Furthermore, they displayed that patients with high microbiome diversity exhibited enhanced T cell memory and natural killer cell signatures in response to anti–PD-1 therapy [97]. The gut microbiota even might play a role in the development of resistance to immunotherapy. The study of Routy et al. discovered an association between a beneficial clinical response on immunotherapy and the relative abundance of *Akkermansia muciniphila*. These results were based on metagenomic analyses of patient stool samples that were taken at diagnosis [98]. *Akkermansia muciniphila* is a well-studied anaerobic bacterium which is specialized in mucus degradation and is associated with human health [99]. To validate these results, fecal microbiota transplantations (FTMs) were performed in mice. The fecal microbiota from cancer patients who developed resistance to immunotherapy was transplanted into germ-free or antibiotic-treated mice. After the FTM, mice were treated with an oral supplementation of *Akkermansia muciniphila*, re-establishing the response to PD-1 based immunotherapy. We also want to highlight that antibiotics can inhibit the clinical benefit of immunotherapy in advanced cancer patients [98]. Researchers treated patients (*n* = 121 renal cell carcinoma (RCC) and 238 NSCLC patients) with antibiotics 30 or 60 days before the start of immunotherapy. In RCC patients, the use of antibiotics was associated with an increased risk of primary progressive disease (PD) (75 versus 22%, *p* < 0.01), shorter PFS (median 1.9 versus 7.4 months) and shorter OS (median 17.3 versus 30.6 months) versus no use of antibiotics. In NSCLC patients, the use of antibiotics was also associated with primary PD (52% versus 43%), but decreased PFS (median 1.9 versus 3.8 months) and OS (median 7.9 versus 24.6 months). The data suggest that the modulation of antibiotic-related gut microbiota composition may be a strategy to improve clinical outcomes with immunotherapy [100]. 

However, confirmation of these promising data in larger study cohorts, as well as more insight and knowledge to overcome some (technical) challenges, are needed [101]. Despite the fact that the study of the microbiota is still in its infancy, it is a biomarker with great potential to monitor NSCLC patients receiving immunotherapy.

## 9. The Electronic Nose

Recently, studying air as a non-invasive biomarker in cancer patients has gained more attention. Again, here, consistent with other liquid biopsy-related publications, exhaled air will be considered as a “liquid” biopsy. Exhaled breath consists of thousands of volatile organic compounds (VOCs). VOCs were identified in 1970 and since then, breath analysis has transformed from a relatively unknown area to a high-throughput breath omics research field [102]. Nowadays, more than 3000 different VOCs in human breath have been identified [102,103,104]. These volatile compounds are produced by several metabolic processes within the human body. Since these processes can be induced by or altered due to disease, it is believed that VOCs cause a specific “breath print” for different diseases. The electronic nose (eNose) was developed, aiming to detect those different breath prints or VOC patterns [104,105].

In 2019, de Vries et al. studied the role of breath to distinguish non-responders from responders to nivolumab or pembrolizumab in NSCLC patients. They collected the exhaled breath data of 143 NSCLC patients at baseline. Samples were taken by a metal oxide semiconductor eNose. They reported that the eNose contributed significantly at baseline in differentiating between patients who responded and who did not at 3 months of anti PD-1 treatment. This study revealed that the eNose could be used to predict individual patient response to immunotherapy [106]. Furthermore, a clinical trial (NCT04146064) is ongoing which addresses breath print analysis as a potential predicting factor for response to immunotherapy. In this trial, 425 participants (with NSCLC, melanoma kidney cancer, urothelial carcinoma (UC) and head and neck cancer (H&NC)) will be included. 

Currently, some challenges prevent this technique from being widespread in clinical practice, such as the stability of the VOCs. Furthermore, the lack of standardized sampling and analysis methods needs to be addressed to implement this technique in clinic. After resolving these challenges, it seems that eNose will find its way to routine practice [102].

## 10. Conclusions and Future Perspectives 

Recently, the introduction of immune checkpoint inhibitor agents targeting PD-1 or PD-L1 has radically modified lung cancer care. Since immunotherapy preferentially targets cancer cells it causes less side effects than traditional chemotherapy. However, only ~20% of the NSCLC patients benefit from it. Therefore, it is crucial to identify: (i) predictive biomarkers in order to select patients who will benefit from immunotherapy and (ii) prognostic biomarkers to monitor patients during therapy, which enables the rapid detection of treatment resistance. Currently, PD-L1 tissue testing is used for the selection of responders to immunotherapy, and imaging is used to monitor patients during their disease course. Unfortunately, obtaining a tissue biopsy is not always feasible due to, e.g., the location of the tumor, and the amount of CT scans per patient each year is limited. 

Liquid biopsy, or the tumor-derived material present in body fluids, may overcome these difficulties. The biggest advantage of this technique in comparison to imaging is its minimally invasive character, which allows repetitive sampling. Furthermore, a liquid biopsy sample can be obtained at almost any moment (regardless of, e.g., the location of the tumor or the health status of the patient) which is an advantage compared to the gold standard PD-L1.

In this review we discussed liquid biopsy-based biomarkers that can be used to select NSCLC patients who will benefit from immunotherapy, such as PD-L1-positive CTCs and exomes. Next, we also highlighted minimally invasive prognostic biomarkers, such as: cf tumor DNA (bTMB and cf tumor DNA levels), methylation signatures, miRNAs, the TCR repertoire, the gut microbiota and VOCs. They are likely to eventually be translated to the clinic in the future. Nevertheless, despite its potential, liquid biopsy is still hampered by some limitations. The biggest bottleneck in most studies is the small sample size. Promising biomarkers should be validated in larger patient cohorts. Secondly, liquid biopsy is limited by the lack of standardization and the absence of broadly accepted standard operating procedures. Lastly, detection problems that arise due to the low abundance of most liquid biopsy compounds need to be resolved. Ongoing clinical trials in NSCLC patients on immunotherapy address these challenges and will give us more insight on the future of liquid biopsy as a biomarker. In summary, nowadays, liquid biopsy is a great additional tool for NSCLC patients on immunotherapy, next to the existing techniques (imaging and PD-L1 tissue testing). However, we hope that one day liquid biopsy can replace the current techniques in order to improve the patients comfort and quality of life.

## Figures and Tables

**Table 2:** Overview of ongoing clinical trials in which promising predictive and prognostic liquid biopsy-based biomarkers are investigated in NSCLC patients. Ongoing clinical trials were found at the website of https://www.Clinicaltrials.gov (accessed on 27 November 2020). An advanced search was performed with the words: “(NSCLC OR non-small cell lung cancer) AND (immunotherapy OR PD-L1 or PD-1) in combination with: (cfDNA OR cell-free DNA); (CTC or circulating tumor cell); exosomes; epigenetics; miRNA; (T cell repertoire OR TCR); gut microbiota or (eNose OR breath)”. Results of the completed trials are available, the other trials have not provided their study results on ClinicalTrials.gov yet. BC: breast cancer; CRC: colorectal cancer; H&NC: head and neck cancer; KC: kidney cancer; MC: melanoma cancer; NSCLC: non-small cell lung cancer; PC: pancreas cancer; RC: renal cancer; UC: urothelial carcinoma.

Trial	Cancer Type	Therapy	# Patients	Markers	End Date
NCT02511288	NSCLC	Immunotherapy, targeted therapy	900	cfDNA, miRNA	2026
NCT04107168	MC, NSCLC, RC	Immunotherapy	1.800	microbiome	2025
NCT04146064	H&NC, KC, MC, NSCLC, UC	Immunotherapy	425	eNose	2024
NCT04638751	BC, CRC, NSCLC, PC	Chemotherapy, immunotherapy	4.000	microbiome	2024
NCT03926260	NSCLC	Chemotherapy, immunotherapy, targeted therapy	100	cfDNA	2023
NCT04629027	NSCLC	Immunotherapy	80	CTC, TCR	2023
NCT04636047	NSCLC	Immunotherapy	450	cfDNA (bTMB), TCR	2023
NCT04636775	NSCLC	Immunotherapy	46	microbiome	2022
NCT04427475	NSCLC	Immunotherapy	900	cfDNA, miRNA	2022
NCT03512847	NSCLC	Chemotherapy, immunotherapy	150	cfDNA	2021
NCT03481101	NSCLC	Chemotherapy, immunotherapy	60	cfDNA	2021
NCT04291755	CRC, NSCLC	Immunotherapy	100	microbiome	2021
NCT03178552	NSCLC	Immunotherapy, targeted therapy	660	cfDNA (bTMB)	2021
NCT02827344	NSCLC	Immunotherapy	200	CTC	2021
NCT03892096	BC, CRC, NSCLC	Chemotherapy, immunotherapy, targeted therapy	750	cfDNA	2021
NCT03576937	NSCLC	Chemotherapy, immunotherapy, targeted therapy	210	cfDNA	2020
NCT03986463	NSCLC	Chemotherapy, radiotherapy	30	cfDNA	2020
NCT03373955	NSCLC	Chemotherapy, immunotherapy, targeted therapy	60	cfDNA, TCR	2020
NCT02551211	NSCLC	Immunotherapy	58	cfDNA, TCR	2019
NCT02890849	NSCLC	Immunotherapy, radiotherapy	60	Exosomes, mRNA	2019
NCT01903993	NSCLC	Chemotherapy, immunotherapy	287	cfDNA (bTMB)	Completed
NCT02008227	NSCLC	Chemotherapy, immunotherapy	1.225	cfDNA (bTMB)	Completed

## Data Availability

The data presented in this review are openly available, references are listed below.

## Abbreviations

(b)TMB	(blood) tumor mutational burden
NFE2L2	nuclear factor erythroid-2-related factor-2
ARID1A	AT-rich interacting domain-containing protein 1A gene
ARID1B	AT-rich interacting domain-containing protein 1B gene
BC	breast cancer
BEAMing	beads, emulsions, amplification and magnetics
CDRs	complementarity-determining regions
cf tumor DNA	circulating cell-free tumor DNA
cfDNA	circulating cell-free DNA
CGP	comprehensive genomic profiling
CI	confidence interval
CRC	colorectal cancer
CTCs	circulating tumor cells
CX3CR1	CX3C chemokine receptor 1
CYTIP	Cytohesin 1 Interacting Protein
ddPCR	digital droplet PCR
eDMRs	differentially methylated regions overlapping enhancers
EGFR	epidermal growth factor receptor
eNose	electronic nose
EpCAM	epithelial cell adhesion molecule
EVs	extracellular vesicles
FDA	food and drug administration
FMTs	fecal microbiota transplantations
FOXP1	forkhead box P1
gDNA	genomic DNA
H&NC	head and neck cancer
HR	hazard ratio
ISET	Isolation by SizE of Tumor cells
KEAP1	kelch-like ECH-associated protein 1
KRAS	Ki-ras2 Kirsten rat sarcoma viral oncogene homolog
Mb	mega base
miRNA	microRNA
MSC	miRNA signature classifier
NGS	next-generation sequencing
NSCLC	non-small cell lung cancer
ORR	overall response rate
OS	overall survival
PBMC	peripheral blood mononuclear cell
PD	progressive disease
PD-1	programmed cell death protein 1
PD-L1	programmed cell death ligand 1
pDMRs	differentially methylated regions overlapping promoters
PFS	progression-free survival
PTEN	phosphatase and TENsin homolog
PTGER4	prostaglandin E receptor 4
RCC	renal cell carcinoma
RECIST	response evaluation criteria in solid tumors
RT-PCR	real time-PCR
SEPT9	septin 9
SHOX2	short stature homeobox 2
STK11	serine/threonine kinase 11
TCR	T cell receptor
TEX	tumor-derived exosomes
tTMB	tissue tumor mutational burden
TNFSF8	TNF superfamily member 8
UC	urotherial carcininoma
VOCs	volatile organic compounds
WES	whole exome sequencing
